# Pathways from self-disclosure to medical coping strategy among adolescents with moderate and major depression during the COVID-19 pandemic: A mediation of self-efficacy

**DOI:** 10.3389/fpsyt.2022.976386

**Published:** 2022-09-02

**Authors:** Yan Wu, Jing Shao, Dawei Zhang, Yongna Wang, Shufen Wang, Zhiren Wang, Yanhua Qu, Jianing Gu

**Affiliations:** Beijing HuiLongGuan Hospital, Peking University, Beijing, China

**Keywords:** adolescent depression, self-disclosure, self-efficacy, medical coping mode, mediation

## Abstract

**Background:**

The prevalence of adolescent depression in China during the COVID-19 pandemic is increasing. Self-disclosing depressive emotions could help release stress. Self-disclosure, which is a prerequisite for self-efficacy, can directly contribute to people’s psychological health, and depression and the choice of coping strategy are determined by the level of self-efficacy perceived.

**Purpose:**

We aimed to discuss the relationship between self-efficacy, self-disclosure, and medical coping strategy. Further, we explore the mediation effect of self-efficacy on the influence of self-disclosure on medical coping strategies in adolescents with depression.

**Methods:**

A total of 585 patients aged 11–24 years with moderate and major depression were recruited. All the assessments were completed on the second day after admission, including the General Self-Efficacy Scale (GSE), Distress Disclosure Index (DDI), and Medical Coping Modes Questionnaire (MCMQ). Pearson correlation was performed to explore the relationships of these variables. The bootstrap analysis was used to conduct to assess the mediation effects.

**Results:**

Both direct and indirect effects of self-disclosure on medical coping strategy were found. As predicted, self-efficacy partially mediated the relationship between self-disclosure and medical coping strategy (*b* = 0.0385, 95% CI: 0.0244–0.0538 for *Confrontation*; *b* = –0.0466, 95%CI: –0.0651 to –0.0296 for *Resignation*), respectively. The effect size for *Confrontation* and *Resignation* was 0.2659 and 0.2485, respectively.

**Conclusion:**

Self-efficacy played a partial mediating role in the effect of self-disclosure on medical coping strategies for adolescent depression during the COVID-19 pandemic, and the use of a positive self-disclosure mechanism may be anticipated to promote improved self-efficacy and the use of active coping strategies.

## Introduction

Depression has been more prevalent among adolescents during the worldwide COVID-19 pandemic compared to before (1), when it was as high as 52.4% in China (2), 32% in Peru, and 9% in Vietnam (3), which will cause an increased risk of negative health outcomes (4). The previous study has found that self-disclosure can directly contribute to people’s psychological health (5), and disclosing depressive emotions could help reduce stress and depression (6).

The onset of depression is important in relationships with coping strategies (7). Coping strategy refers to intrapsychic activities, as well as to the communications and behaviors of patients, designed to the decrease of distress caused by illness. As techniques used by patients to deal with disease, coping strategies are generally categorized into two broad types: active strategies and evasive strategies. Active strategies aim at making an active stress response. Evasive strategies mean evading stressful situations (8), and usually entail maladaptive consequences among adolescents (9–11). Compared with healthy people, depressive patients more often utilize strategies based on avoidance and denial, and experience more difficulty in finding positive characteristics in stressful events (12). However, the influence and pathway of self-disclosure among depressive adolescents in terms of adopting coping strategies has been unclear.

The choice of one coping strategy over another is determined by the level of perceived self-efficacy (13). Self-efficacy is a belief in one’s own capacity to face challenges (14), which is a key factor for adolescents’ emotional wellbeing. Low subjective wellbeing may significantly predict increased depressive symptoms (15). Based on Bandura’s social cognitive theory (16), self-efficacy is the faith in one’s ability to perform the behaviors that are necessary to attain a desired goal. Self-efficacy beliefs guide action both directly and indirectly through self-expectations regarding the result of a certain behavior (14). High self-efficacy for dealing with stress can prevent or reduce stress along with its health influences (17). However, the previous study showed that authentic self-disclosure to at least one person is a prerequisite for self-efficacy of psychological adjustment (18). In this situation, the use of a positive self-disclosure behavior may promote improved self-efficacy, the use of active coping strategies, and reduce depression. However, the increase in depression during the COVID-19 pandemic means that many of them need to choose the proper coping strategy to solve their depressive disorders.

**FIGURE 1:**
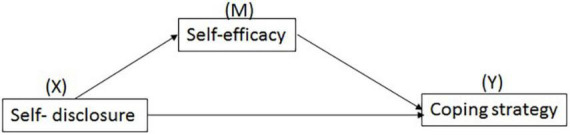
Path model for the mediation effect of self-efficacy on the influence of self-disclosure on medical coping strategy of adolescents with depression.

However, much of the existing literature focuses on the relationship among self-disclosure, self-efficacy, coping strategies, and depression. Therefore, the present study was designed to discuss the relationship between self-disclosure, self-efficacy, and medical coping strategies and to explore the mediation effect of self-efficacy on the influence of self-disclosure on medical coping strategies in adolescents with depression (Figure 1). The outcomes of the present study may ultimately help adolescents to deal with their situations more positively. Moreover, we provide foundational data for developing nursing interventions to improve depression in adolescents before antidepressant therapy.

## Materials and methods

### Participants

Data of the present study were collected through a cross-sectional research in Beijing, China between June 2020 and July 2021 during the COVID-19 pandemic. Based on the literature recently published (19), the age range of description of adolescence was 10–24 years. A total of 585 patients aged 11–24 years old with moderate and major depression were recruited. All participants were students without married, and they lived with parents and schoolmates.

All participants met the eligibility criteria, i.e., meeting the diagnostic criteria for depression according to the Diagnostic and Statistical Manual of Mental Disorders, 4th Edition. The exclusion criteria for the patients were rigorous, such as neurological disorders, head trauma, intellectual disability, bipolar disorder, and other psychiatric disorders.

Sociodemographic data, including age and years of education, were collected. Clinical data, including disease course, was obtained according to medical records, and self-report and confirmed by the next of kin and family members.

Approval for the present study was obtained from the Ethics Committee of Beijing HuiLongGuan Hospital which was in accordance with the principles in the Declaration of Helsinki. Written informed consent was obtained from all patients or their guardians before study enrollment.

### Measures

In the present study, all the assessments were completed for all patients on the second day after admission, including the General Self-Efficacy Scale (GSE), Distress Disclosure Index (DDI), and Medical Coping Modes Questionnaire (MCMQ).

### General self-efficacy scale

The GSE is a 10-item self-report scale which assesses general self-efficacy as a prospective and operative construct. Each item is scored from 1 (not at all true) to 4 (totally true). The total score ranges from 10 to 40, with higher scores showing higher self-efficacy (20). The GSE assesses optimistic self-belief in dealing with the demands, tasks, and challenges of life in general (21). Cronbach’s α coefficient was 0.84 (22).

### Distress disclosure index

The DDI is a 12-item self-report assessment of one’s tendency to disclose personally distressing thought, a construct termed distress disclosure. DDI measures individual typical disclosure of distressing thoughts, personal difficulties, and distasteful emotions across time and circumstances. Participants assessed their agreement with each item from 1 (strongly disagree) to 5 (strongly agree). As a result, the DDI is useful to measure individual differences in emotional self-disclosure which is relevant to the counseling or seeking support process. Cronbach’s α coefficient was 0.93 (23).

### Medical coping modes questionnaire

The Chinese version of the MCMQ which is aimed to measure three coping strategies that participants may utilize when facing life-threatening diseases, namely, *Confrontation, Avoidance*, and *Resignation*, contains 20 items (1 item was added to keep the original meaning of the scale when it was translated into Mandarin Chinese). For each item, individuals choose the response from the 4 choices that best reflects his or her experience (1 = none, 2 = a few, 3 = quite a few, 4 = a lot). Eight of the 20 items are reverse-scored. Subscale scores are attained by summing each item scores (range = 8–32 for *Confrontation*, 7–28 for *Avoidance*, 5–20 for *Resignation*). Higher scores show that an individual has more behaviors described by that specific coping scale when handle medical incidents. The MCMQ-C was used in the current study, and a good internal consistency was shown in the subscales, with Cronbach’s α coefficients as follows: *Confrontation*, 0.74; *Avoidance*, 0.73; and *Resignation*, 0.83 (24).

### Statistical analyses

Descriptive statistics were used to calculate the frequency of characteristics of the samples. Continuous variables were described by mean and standard deviation (*SD*), and categorical variables by frequency and percentage (%). Correlation analysis was performed using partial correlation.

Mediation analysis was computed for self-disclosure as an independent variable, medical coping mode as a dependent variable (*Y*), and self-efficacy as a possible mediator (*M*). The PROCESS v3.5 for SPSS (25) was used with bootstrapping with coefficients estimated from 5,000 bootstraps. A simple mediational approach was conducted using Model 4 with 5,000 bootstraps with education and disease course as covariates. The output mediation models were explained as follows: *b (YX)* is the total effect of the independent variable *X* on the dependent variable *Y*; *b (MX)* is the effect of the independent variable on the assumed mediator *M*; *b (YM.X)* is the effect of the mediator on the dependent variable, excluding the independent variable; and *b (YX.M)* is the direct effect of the independent variable on the dependent variable except for the mediator. The indirect effect of *X* on *Y* through *M* was estimated. The ratio of the indirect effect to the total effect was treated as the effect size for mediation (26). The statistical analyses were conducted using IBM SPSS Statistics for Windows (version 23.0; IBM Corp., Armonk, NY). *P*-value < 0.05 was considered significant.

## Results

### Descriptive statistics

A total of 585 participants were included in the analysis, with an average of 17.04 ± 3.23 years old. As shown in Table 1, male participants were 135 (23.08%) and the rest of participants were female (*n* = 450, 76.92%). There were 346 participants of age ≤ 18 years (14.75 ± 1.55), while 239 participants were aged >18 years (20.36 ± 1.88). The average years of education is 11.04 ± 3.23. The average disease course is 2.18 ± 1.67 years. The distributions of DDI, GSES, *Confrontation*, *Avoidance*, and *Resignation* of MCMQ were 31.73 ± 9.64, 20.07 ± 6.44, 17.53 ± 4.07, 16.40 ± 3.10, 12.43 ± 3.98 (Table 1).

**TABLE 1:** The demographic and psychotypical characteristics of adolescents with depression on admission (*n* = 585).

Variables	Number/Mean ± *SD*
Gender (Male: Female)	135:450
Age (years)	17.04 ± 3.23
≤18 (*n* = 346)	14.75 ± 1.55
>18 (*n* = 239)	20.36 ± 1.88
Education (years)	11.04 ± 3.23
Disease course (years)	2.18 ± 1.67
DDI	31.73 ± 9.64
GSES	20.07 ± 6.44
MCMQ	
Confrontation	17.53 ± 4.07
Avoidance	16.40 ± 3.10
Resignation	12.43 ± 3.98

GSES, general self-efficacy scale; DDI, Distress Disclosure Index; MCMQ, medical coping modes questionnaire; SD, standard deviation.

All data were reported as mean ± SD using Mann-Whitney sum tests.

### Correlation analysis

Table 2 shows the correlation between the variables from the multivariate analysis with education and disease course as covariates. The scores of DDI and GSES were both correlated with the scores of confront and resignation coping strategies, respectively (*r* = 0.341/-0.441 and *r* = 0.347/-0.430, all *p* < 0.001). The scores of DDI were correlated with the scores of GSES (*r* = 0.349, *p* < 0.001). However, neither DDI nor GSES was correlated with avoidance coping strategy (both *p* > 0.05).

**TABLE 2:** Correlation among psychotypical characteristics of adolescents with depression before (*n* = 585).

Variables	DDI		GSES	
	r	*p*	r	*p*
GSES	0.349	<0.001*	–	–
MCMQ				
Confrontation	0.341	<0.001*	0.347	<0.001*
Avoidance	–0.055	0.182	0.063	0.126
Resignation	–0.441	<0.001*	–0.430	<0.001*

GSE, general self-efficacy; DDI, Distress Disclosure Index; MCMQ, medical coping modes questionnaire.

Partial correlation was used to calculate the correlation between the variables with education and disease course as covariates, *p < 0.05.

### Mediation analysis for self-efficacy

**FIGURE 2:**
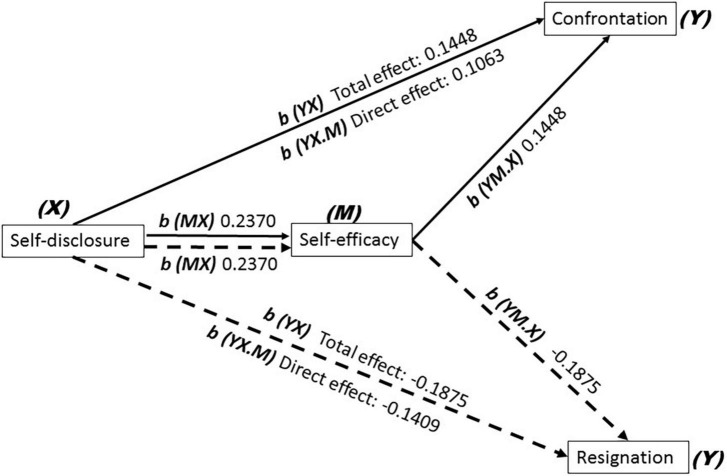
On admission, *Confrontation* and *Resignation* coping strategies of adolescents with depression were, respectively used as *Y*, and mediation analysis results showed that *Model b (YX)* (total effect, *b* = 0.1448 for *Confrontation*, and *b* = –0.1875 for *Resignation*; both *p* < 0.001), *Model b (MX)* (*X* – > *M*, for both *Confrontation* and *Resignation, b* = 0.2370, *p* < 0.001), *Model b (YM.X)* (*M* – > *Y*, *b* = 0.1448 for *Confrontation*, and *b* = –0.1875 for *Resignation*; both *p* < 0.001), *Model b (YX.M)* (*X* – > *Y*, *b* = 0.1063 for *Confrontation* and *b* = –0.1409 for *Resignation*; both *p* < 0.001). The coefficient of indirect of *Confrontation* and *Resignation* was 0.0385 and –0.0466 (*X* – > *M* – > *Y*, for *Confrontation* and for *Resignation*).

*Confrontation* and *Resignation* coping strategies were, respectively used as *Y*, and mediation analysis results showed that *Model b (YX)* (total effect, *b* = 0.1448, *t* = 8.7446, 95%CI: 0.1123–0.1774 for *Confrontation* and *b* = –0.1875, *t* = –11.8580, 95%CI: –0.2185 to –0.1565 for *Resignation*; both *p* < 0.001), *Model b (MX)* (*X* – > *M*, for both *Confrontation* and *Resignation*, **b** = 0.2370, *t* = 8.9899, 95%CI: 0.1852–0.2887 and both *p* < 0.001), *Model b (YM.X)* (*M* – > *Y*, *b* = 0.1448, t = 8.7446, 95%CI: 0.1123–0.1774 for *Confrontation* and *b* = –0.1875, *t* = –11.8580, 95%CI: –0.2185 to –0.1565 for *Resignation*; both *p* < 0.001), and *Model b (YX.M)* (*X* – > *Y*, *b* = 0.1063, *t* = 6.2206, 95%CI: 0.0727–0.1399 for *Confrontation* and *b* = –0.1409, *t* = –8.8305, 95%CI: –0.1726 to –0.1096; both *p* < 0.001 for *Resignation*). The coefficient of indirect of *Confrontation* and *Resignation* was 0.0385 and –0.0466 (*X* – > *M* – > *Y*, 95%CI: 0.0244–0.0538 for *Confrontation* and 95%CI: –0.0651 to –0.0296 for *Resignation*). The ratio of indirect effect/total effect for *Confrontation* and *Resignation* was 0.2659 and 0.2485, respectively. These outputs identified the partial mediation effect of self-efficacy *(M)* on *Confrontation* and *Resignation (Y)* (Figure 2). However, there was no mediation effect of self-efficacy *(M)* on *Avoidance* (*p* > 0.05).

These results above partially support the hypothesis that self-efficacy which mediated medical coping strategies with self-disclosure remains a predictor.

## Discussion

To the best of our knowledge, much of the existing literature focuses on the relationship among self-disclosure, self-efficacy, coping strategies, and depression, but there are few studies of the mediation of self-efficacy between self-disclosure and medical coping strategy in adolescents with depression. The present study is the first to examine the relationship between self-disclosure and medical coping strategy during the COVID-19 pandemic. Further, we explored the possible pathways underlying this association concerning depressive adolescents’ self-efficacy by constructing a mediation model, and offering a more comprehensive picture of self-disclosure-coping strategy pathways. Understanding the coping strategies of depressive adolescents during the COVID-19 pandemic and its underlying psychological mechanisms are crucial for the effective intervention of adolescents with depression before medication and are beneficial for establishing a team of high-quality nurses’ and consultants’ to fight future public health emergencies.

The primary finding in the present study revealed that self-disclosure has a direct effect on medical coping strategies, mainly the choice of *Confrontation* and *Resignation*. Adolescents would need direct contact with the outside world, and long-term staying at home would have a great influence on emotion. The prolonged home confinement decreased social interactions with peers for children and adolescents (27, 28). They are compelled to lock themselves down comprehensively, and find it harder to articulate their feelings during the COVID-19 pandemic, which has caused a persistent influence on the mental health of children and adolescents and ultimately contribute to the occurrence of depressive symptoms (29). It was found in a domestic study that the occurrence of emotional difficulties in children and adolescents is much higher than that before the pandemic (30).

Previous studies have shown that disclosing depressive emotions could help release depression (6). Self-disclosure refers to the course by which persons open themselves up to others (31), and has long been identified as a key coping behavior in therapeutic situation (32). Several studies have demonstrated self-disclosure as a prerequisite for healthy adjustment (33). An individual with positive self-disclosure can have effective communication with the outside world (30). On the other hand, non-disclosure is often used to refer to non-expression, inhibition, or topic avoidance. A growing body of research documents that non-disclosure is associated with poor psychological adjustment and increased distress (34). The social-cognitive processing model of adjustment to stressors illustrates the impact of non-disclosure on emotional distress (34), which may undermine an individual’s ability to confront others. A previous study has demonstrated the negative impact of non-disclosure of stress-related thoughts and feelings on depressive symptoms (35, 36). Disclosing stressful events and discussing related thoughts and feelings with others may present the opportunity to integrate the cognitive discrepancy by receiving new information and/or attaining appropriate support (37) which, in turn, may promote emotional adjustment.

Based on the definition of the coping strategies of stress and the coping theory, self-disclosure can be considered as a coping strategy when people disclose their problems and need to attain social support (38). Coping refers to how people try to deal with traumatic events or everyday stressful situations. It plays a crucial role in adolescence, which is a period during which individuals manage new challenges that can stand for Mayordomo-Rodríguez et al. (39). The previous study has reported that positive coping strategies were connected to a greater feeling of control over occurrences and the search for useful information to cope with difficulties (40). According to the stress-buffering model (41), a positive coping style offers buffering effects in the path of disease and psychological distress (42). Some researchers have confirmed that adopting a positive and effective coping strategy can reduce the psychological pressure on them (43), thus improving depression.

Generally, *Confrontation* is a positive coping strategy, while *Avoidance* and *Resignation* are negative coping strategies (44). The use of active coping is supportive of mental health (45), which helps prevent depression (46). Avoidance coping strategy is oriented toward denying, minimizing, or else avoiding dealing directly with stressful requests and is closely linked to distress, and the use of *Avoidance*-coping strategy has been related to relapse among patients treated for depression (47). However, the course of depression among the participants was 2.18 ± 1.67 years, and there were fewer patients with recurrent depression. Therefore, the effect of self-disclosure on *Avoidance* was not found in the present study. The literature suggests that *Resignation* is related to depression (48, 49). *Resignation* as a negative orientation has a negative influence on stress and has been associated with an increase in depressive symptoms and poorer wellbeing, and reflects factors related to hopelessness and giving up (49). Consequently, self-disclosure was positively and negatively correlated with *Confrontation* and *Resignation*, respectively, in our study, which will help us understand the direct effect of self-disclosure on medical coping strategy.

The secondary findings demonstrated that self-efficacy partially mediated the relationship between self-disclosure and medical coping strategies. The previous study showed that the prerequisite for self-efficacy is authentic self-disclosure (18). A high self-disclosure may mean a high level of self-efficacy (50, 51). Self-efficacy refers to a person’s faith about their ability to mobilize courses of action needed to achieve desired personal goals (52). Further, it is considered an influential motivational, cognitive, and affective determinant of student action, with a significant impact on their involvement, effort, persistence, self-regulation, and achievement (8). These features make self-efficacy a vital variable in controlling stress (8), and it is a protective factor against the influence of day-to-day stressors at school (53, 54). Moreover, a high level of self-efficacy is conducive for patients to take a positive coping strategy (55). *Confrontation* has been shown to be supportive of mental health (45) to protect against depression (46), and *Resignation* has been associated with an increase in depressive symptoms (49), respectively. In the present study, we also found that GSES was positively and negatively correlated with *Confrontation* and *Resignation*, respectively. All the literature above can explain fully the partial mediation of self-efficacy between self-disclosure and medical coping strategy.

Other findings of our current study included that the effect size for *Confrontation* and *Resignation* was 0.2659 and 0.2485, respectively. The previous study has reported that females had more frequent use of support-seeking and active coping strategies than males (56). However, the number of females in our study was more than that of males, therefore, it was reasonable that the effect size for *Confrontation* was higher than that for *Resignation* in our study.

There are some limitations to the present study. First, although the number of the recurrent and the first-episode depression, and the assessment of depressive symptoms were not provided, all participants who were diagnosed with moderate and major depression needed antidepressant medication, which means that their psychological state was assessed without knowing depressive symptoms to decrease the dependence on symptom scores and treatment or not, and there is no need to excessively increase the workload of nurses. Secondly, the effect of age range and gender differences on the pathway of the mediation was not discussed here, because what the whole children and adolescents were analyzed together will help us to take measurements of the effective intervention of the population. Thirdly, although the mediating effect size was very small in the present study, the previous study has reported that the ratio of the indirect effect to the total effect has been a popular measure in mediation analysis (57), and the ratio of indirect effect/total effect for *Confrontation* and *Resignation* was 0.2659 and 0.2485, respectively. Therefore, the mediation effect had been still reported. Finally, although our cross-sectional study did not determine the causality, and a longitudinal study design is better suited for confirming the relationship, the present study did provide evidence for medical coping strategies chosen in adolescents with depression.

## Conclusion

In the present study, self-efficacy indeed played a partial mediating role in the effect of self-disclosure on medical coping strategies for adolescent depression during the COVID-19 pandemic. The use of a positive self-disclosure mechanisms may be necessary to promote improved self-efficacy and the use of active coping strategies.

## Data availability statement

The raw data supporting the conclusions of this article will be made available by the authors, without undue reservation.

## Ethics statement

The study was approved by the Institutional Review Board of the Beijing HuiLongGuan Hospital. Written informed consent to participate in this study was provided by the participants or their legal guardian/next of kin.

## Author contributions

YaW designed the study, led the statistical analyses, and drafted the manuscript. JS provided clinical coordination. DZ and YoW finished the assessments. SW collected the clinical data. YQ and JG provided the input data. YaW and ZW secured funding for the present study. All authors approved the final manuscript for submission.
